# Serum folate associated with nonalcoholic fatty liver disease and advanced hepatic fibrosis

**DOI:** 10.1038/s41598-023-39641-1

**Published:** 2023-08-09

**Authors:** Hao-Kai Chen, Jing Luo, Xiu-Juan Li, Wan-Zhe Liao, Yu-Qi Hu, Xu-Guang Guo

**Affiliations:** 1 Department of Clinical Laboratory Medicine, Guangdong Provincial Key Laboratory of Major Obstetric Diseases; Guangdong Provincial Clinical Research Center for Obstetrics and Gynecology; The Third Affiliated Hospital of Guangzhou Medical University, Guangzhou, China; 2 Department of Clinical Medicine, The Third Clinical School of Guangzhou Medical University, Guangzhou, China; 3 Department of Clinical Medicine, The Nanshan College of Guangzhou Medical University, Guangzhou, China; 4 Guangdong Provincial Key Laboratory of Major Obstetric Diseases, The Third Affiliated Hospital of Guangzhou Medical University, Guangzhou, China; 5 Key Laboratory of Reproduction and Genetics of Guangdong Higher Education Institutes, The Third Affiliated Hospital of Guangzhou Medical University, Guangzhou, China; 6 Guangzhou Key Laboratory for Clinical Rapid Diagnosis and Early Warning of Infectious Diseases, King Med School of Laboratory Medicine, Guangzhou Medical University, Guangzhou, China

**Keywords:** Endocrine system and metabolic diseases, Non-alcoholic fatty liver disease

## Abstract

The role played by serum folate in the progression of nonalcoholic fatty liver disease (NAFLD) remains controversial. The purpose of this study was to investigate the association of serum folate with NAFLD and advanced liver fibrosis (AHF). We conducted a cross-sectional study with 5417 participants using 2011–2018 NHANES data. Multiple logistic regression analysis and propensity score matching analysis were used to investigate the association of serum folate with NAFLD and AHF. In the completely adjusted model, participants in the high serum folate group had a 27% (OR 0.73, 95% CI 0.62, 0.87, p = 0.0003) and 53% (OR 0.47, 95% CI 0.35, 0.63, p < 0.0001) lower odds of suffering from NAFLD and AHF, respectively, compared to the low serum folate group. The similar results in propensity score matching further validated the above association. Stratified analysis showed that the negative correlation of serum folate with NAFLD and AHF demonstrated a broad consistency across populations. The results of this study indicate that higher serum folate level was associated with lower odds of NAFLD and AHF among US adults. Further prospective studies are necessary due to the limitations of cross-sectional studies.

## Introduction

Nonalcoholic fatty liver disease (NAFLD) is a widespread metabolic liver disease with excessive fat deposits in the liver, excluding other factors of liver injury and significant alcohol consumption^1^. Intracellular triglycerides are present in more than 5% of hepatocytes in NAFLD patients^1^. Advanced liver fibrosis (AHF) usually develops from abnormal proliferation of intrahepatic connective tissue due to chronic liver injuries such as NAFLD and is thought to be significantly associated with cirrhosis or liver failure^2^. In the past several decades, the incidence of NAFLD has grown rapidly and has become one of the prime causes of liver disease globally^3^. It is estimated that the overall prevalence rate of NAFLD globally is 32.4%, showing obvious sex differences, with males having a significantly higher prevalence rate than females^4^. Metabolic disorders such as central obesity, dyslipidemia, hypertension, hyperglycemia, and continuous liver function abnormalities are closely related to NAFLD^5^. Studies have shown that NAFLD is an independent risk factor for type 2 diabetes, cardiovascular disease, and other liver-related complications^6^. The association between NAFLD and hepatocellular liver cancer is becoming increasingly apparent as the number of obese and type 2 diabetic patients increases globally^7^. In summary, NAFLD and AHF pose a serious burden on human health. Although efforts are being made to find drugs to treat NAFLD and AHF, there is no specific drug licensed that can completely reverse NAFLD or AHF^8^. Therefore, it is particularly important to explore new intervention mechanisms, therapeutic agents and targets for NAFLD and AHF.

Folate is a water-soluble vitamin, and naturally occurring folate is a combination of pteroic acid and glutamic acid. Folate deficiency causes megaloblastic anemia and hyperhomocysteinemia and increases the risk of atherosclerosis, thrombosis and hypertension^9^. A past cohort study from China showed that low serum folate levels contribute to NAFLD risk^10^. According to a study by Tripathi et al. in mice, dietary intake of folic acid improved liver tissue status in nonalcoholic steatohepatitis^11^. In addition, their study showed that serum folate may play an important role in preventing or delaying disease progression in NASH as well as reversing liver inflammation and fibrosis^11^. A recent study adopted both meta-analysis and Mendelian randomization analysis to demonstrate a negative association between serum folate and the risk of NAFLD^12^. However, past evidence has not always been consistent. Two cross-sectional studies based on US populations associated with NAFLD both claimed not to have observed a correlation between serum folate or dietary folate and NAFLD^13,14^. Although the major subject of their study was not serum folate, it does suggest that the association of serum folate with NAFLD is still controversial. In addition, studies on serum folate and AHF are still limited.

To our knowledge, there are no epidemiological studies on the association between serum folate and NAFLD or AHF in US adults. Therefore, we conducted a cross-sectional study including 5417 participants based on NHANES 2011–2018, aiming to investigate the association of serum folate with NAFLD and AHF. We believe that this study will provide new ideas for the treatment and management of NAFLD.

## Materials and methods

### Data sources and study design

The sample for this study was obtained from the 2011–2018 National Health and Nutrition Examination Survey (NHANES) data. NHANES is a nationally representative cross-sectional research program on nutrition and health designed to collect information on demographics, dietary assessments, health interviews, physical examinations, and laboratory tests in the noninstitutionalized population of the United States. Demographic, health status, and laboratory data of participants were obtained by trained professionals through questionnaires, health interviews, and laboratory tests. The dietary status of participants was obtained through a 24-h dietary recall over two days, and physical examinations and blood samples were collected in the mobile examination center (MEC).

**Figure 1:**
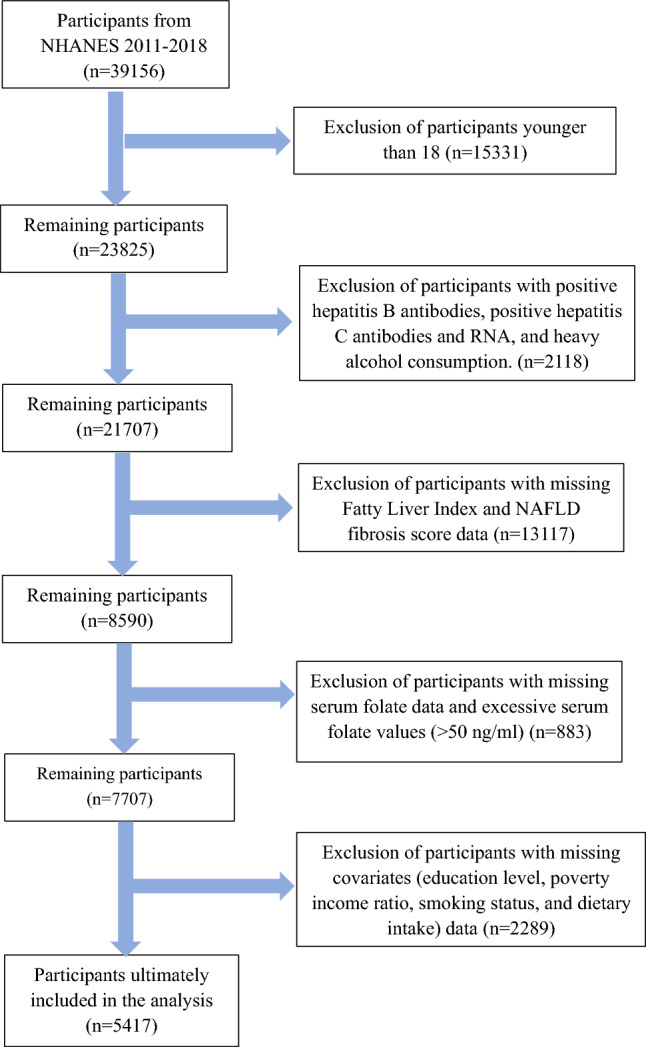
Flow chart for inclusion and exclusion of participants.

Participant data marked as missing, refused, and did not know in the NHANES database were considered missing data and excluded manually. To include participants meeting the study objectives, we developed exclusion criteria: (1) age < 18 years; (2) positive for hepatitis B antibody, hepatitis C antibody or hepatitis C RNA; (3) heavy alcohol consumption (> 30 g/day for males, > 20 g/day for females); (4) missing data for fatty liver index, NAFLD fibrosis score, and serum folate; (5) abnormally high serum folate levels (> 50 ng/mL); and (6) missing data for covariates such as education, poverty income ratio, smoking status, and dietary intake. The process of inclusion and exclusion is shown in Fig. 1. A total of 5417 participants were eventually included in the analysis.

### Measurement of serum folate

Measurement of serum folate was performed by isotope-dilution high-performance liquid chromatography coupled to tandem mass spectrometry (LC‒MS/MS). At the beginning of the measurement, 150 μL of serum sample was combined with ammonium formate buffer as well as an internal standard mixture. Subsequently, samples were extracted using automated 96-probe solid phase extraction (SPE) with 96-well phenyl SPE plates. Folate forms were separated using isocratic mobile phase conditions and measured by LC‒MS/MS.

### Definition of NAFLD and AHF

The fatty liver index (FLI) was used to define NAFLD in this study. FLI is a widely used surrogate marker to predict the risk of NAFLD and is recommended by European guidelines for the management of NAFLD^15,16^. Participants with an FLI score greater than or equal to 60 were considered to have NAFLD^17^. The NAFLD fibrosis score (NFS) is a nondiffusion system for identifying nonalcoholic fatty liver fibrosis, and participants in this study with NFS > 0.676 were considered to have AHF^18^. It is important to note that the definitions of both NAFLD and AHF are based on non-invasive scores. The equations for FLI and NFS are shown below^17,18^.

$${\text{FLI}} = \left( {{\text{e}}^{{0.{953} \times {\text{loge}}\left( {{\text{TG}}} \right) + 0.{139} \times {\text{BMI}} + 0.{718} \times {\text{loge}}\left( {{\text{GGT}}} \right) + 0.0{53} \times {\text{WC}} - {15}.{745}}} } \right)/\left( {{1} + {\text{e}}^{{0.{953} \times {\text{loge}}\left( {{\text{TG}}} \right) + 0.{139} \times {\text{BMI}} + 0.{718} \times {\text{loge}}\left( {{\text{GGT}}} \right) + 0.0{53} \times {\text{WC}} - {15}.{745}}} } \right) \times {1}00;$$

NFS =  − 1.675 + 0.037 × age + 0.094 × BMI + 1.13 × impaired fasting glycemia or diabetes (yes = 1, no = 0) + 0.99 × AST/ALT ratio − 0.013 × platelet − 0.66 × albumin.

TG, Triglycerides; GGT, gamma-glutamyl transferase; WC, waist circumference.

### Covariates

Since the results of the study may be influenced by multiple factors, we included age, sex, race, education level, poverty income ratio (PIR), BMI, smoking status, work activity status, recreational activity status, dietary energy, protein, alcohol, folate intake, hypertension status, diabetes status and biochemical indicators, including total cholesterol and HDL cholesterol, as covariates of the study. Five racial classifications, including Mexican American, Other Hispanic, Non-Hispanic Black, Non-Hispanic White, and Other races, were used to define the race variable. Education level is classified as < high school, high school, and > high school. The poverty income ratio was categorized as < 1, 1–3, and > 3. BMI was classified as < 18.5 (underweight), 18.5–24.9 (healthy weight), or > 25 (overweight or obesity)^19^. Smoking status was categorized as never, former, and now. Four scales, including no, vigorous, moderate and both, were used to evaluate the work or recreational activities status of participants. The dietary intake status used the sum of the dietary intake of the first and second day. Participants using hypertensive medications or with past/current diagnosis of hypertension were diagnosed with hypertension. Diabetes status was grouped as yes, no, impaired fasting glucose, impaired glucose tolerance based on hypoglycemic medication status, diabetes diagnosis status, glycated hemoglobin, and fasting glucose. All covariate data in this study were obtained from the NHANES website (https://www.cdc.gov/nchs/nhanes/index.htm).

### Statistics analysis

In participant characterization, continuous variables are expressed as "mean ± standard deviation" or "median (interquartile range)". The median (interquartile range) is used when the standard deviation of a continuous variable is greater than half of the mean. Number and percentage (%) were used to describe the categorical variables. The χ^2^ test and Kruskal‒Wallis test were used to evaluate the statistical significance of categorical and continuous variables.

Multiple logistic regression analysis was used to evaluate the association of serum folate with NAFLD or AHF, and adjusted models were constructed based on the included covariates. No variables were adjusted in the crude model. Model 1 was adjusted by age, sex, race, education level, and PIR. Model 2 further adjusted for total cholesterol, HDL cholesterol, hypertension status and diabetes status. Model 3 is a fully adjusted model, with the addition of adjusted smoking status, work activities status, recreational activities status, dietary energy intake, dietary protein intake, dietary folate intake and dietary alcohol intake. In multiple logistic regression, serum folate was trisected into low (1.8–12.6 ng/mL, n = 1806), medium (12.7–20.5 ng/mL, n = 1789) and high (20.6–49.9 ng/mL, n = 1822) groups, and the low serum folate group was used as the reference group. We calculated the z score of serum folate and reported the odds ratios (OR) of NAFLD and AHF with each standard deviation (SD) increase in serum folate. Subsequently, we visualized the association by plotting a smoothed fit curve based on adjusted model 3 (ln-transformed data).

Propensity score matching (PSM) has been widely used to control for selection bias in observational studies. In this study, based on a 1:1 nearest neighbor matching algorithm, we used PSM to match participants with NAFLD or AHF to controls. Confounding factors, including age, sex, race, education level, poverty income ratio (PIR), smoking status, work activity status, recreational activity status, dietary energy, protein, alcohol, folate intake, hypertension status, diabetes status, total cholesterol, and HDL cholesterol, were chosen for matching. In addition, stratified analyses were constructed based on age, sex, race, education, and PIR to examine the stability of the association of serum folate (per SD increment) with NAFLD or AHF. For all analyses, the level of statistical significance was determined to be 2-sided p < 0.05, and 95% confidence intervals were calculated in this study. Using appropriate strata, clusters, and weights in the statistical analysis process to illustrate the complex multistage stratified sampling design of NHANES. The researchers used the statistical packages R (The R Foundation; http://www.r-project.org; version 3.6.3) and Empower Stats software (www.empowerstats.net, X&Y solutions, Inc. Boston, Massachusetts) to perform the data processing.

### Ethics statement

The authors are accountable for all aspects of the work in ensuring that questions related to the accuracy or integrity of any part of the work are appropriately investigated and resolved. The study was conducted in accordance with the Declaration of Helsinki (as revised in 2013). All information from the NHANES program is available and free for public, so the agreement of the medical ethics committee board was not necessary.

## Results

### Baseline characteristics of participants based on NAFLD stratification

NHANES data from 2011 to 2018 were used for this study, with a total of 5417 participants included in the analysis. The baseline characteristics of the participants stratified based on NAFLD are shown in Table 1. Based on the FLI, NAFLD was confirmed in 2361 participants. Compared to participants without NAFLD, those with NAFLD were more likely to be older, male, non-Hispanic White, less educated, have a lower PIR, be past or current smokers, have more intense work activity, lack recreational activity, have hypertension or diabetes, and have a higher waist circumference or BMI. In terms of biochemical indicators, participants with NAFLD had higher levels of GGT, triglycerides, total cholesterol, AST, and ALT. However, there were no significant differences observed in terms of dietary energy and protein intake. More importantly, serum folate levels were lower in participants with NAFLD [33.98 (24.01–50.06) vs 38.51 (26.27–55.72), p < 0.001].

**Table 1:** Baseline characteristics of participants based on NAFLD stratification.

Characteristic	NAFLD		p-value
	No	Yes	
N	3056	2361	
Demographics			
Age (year), mean ± SD	47.84 ± 18.12	51.06 ± 15.97	< 0.001
Sex			
Male	1318 (43.13%)	1133 (47.99%)	
Female	1738 (56.87%)	1228 (52.01%)	
Race			< 0.001
Mexican American	342 (11.19%)	388 (16.43%)	
Other Hispanic	302 (9.88%)	263 (11.14%)	
Non-Hispanic White	1188 (38.87%)	1024 (43.37%)	
Non-Hispanic Black	613 (20.06%)	484 (20.50%)	
Other race—including multi-racial	611 (19.99%)	202 (8.56%)	
Education level			< 0.001
< High school	555 (18.16%)	513 (21.73%)	
High school	613 (20.06%)	575 (24.35%)	
> High school	1888 (61.78%)	1273 (53.92%)	
PIR			< 0.001
< = 1	628 (20.55%)	549 (23.25%)	
1–3	1213 (39.69%)	1059 (44.85%)	
> 3	1215 (39.76%)	753 (31.89%)	
Behavioral characteristics			
Smoking status			< 0.001
Never	1929 (63.12%)	1285 (54.43%)	
Now	488 (15.97%)	421 (17.83%)	
Former	639 (20.91%)	655 (27.74%)	
Work activities status			0.048
No	1813 (59.33%)	1312 (55.57%)	
Moderate	662 (21.66%)	559 (23.68%)	
Vigorous	118 (3.86%)	94 (3.98%)	
Both	463 (15.15%)	396 (16.77%)	
Recreational activities status			< 0.001
No	1349 (44.14%)	1359 (57.56%)	
Moderate	858 (28.08%)	624 (26.43%)	
Vigorous	279 (9.13%)	122 (5.17%)	
Both	570 (18.65%)	256 (10.84%)	
Dietary characteristics			
Dietary energy intake (kcal), Mean ± SD	3934.68 ± 1481.90	3989.11 ± 1616.94	0.198
Dietary protein intake (mg), mean ± SD	156.71 ± 66.36	160.26 ± 70.70	0.058
Dietary folate intake (mcg), median (Q1-Q3)	710.00 (500.00–985.25)	673.00 (476.00–928.00)	0.012
Related disease conditions			
Hypertension status			< 0.001
No	2084 (68.19%)	1053 (44.60%)	
Yes	972 (31.81%)	1308 (55.40%)	
Diabetes status			< 0.001
No	2276 (74.48%)	1075 (45.53%)	
Yes	352 (11.52%)	788 (33.38%)	
IGT	210 (6.87%)	194 (8.22%)	
IFG	218 (7.13%)	304 (12.88%)	
Anthropometric measurements			
Waist circumference (cm), mean ± SD	89.16 ± 9.84	114.25 ± 13.50	< 0.001
BMI (kg/m^2^), mean ± SD	25.13 ± 3.68	35.23 ± 6.55	< 0.001
Biochemical indicators			
GGT (IU/L), median (Q1–Q3)	16.00 (12.00–21.00)	24.00 (18.00–38.00)	< 0.001
Triglycerides (mg/dL), median (Q1–Q3)	78.00 (56.00–108.00)	130.00 (91.00–187.00)	< 0.001
Total cholesterol (mg/dL), mean ± SD	185.00 ± 39.41	194.28 ± 41.41	< 0.001
HDL cholesterol (mg/dL), mean ± SD	58.13 ± 15.51	46.82 ± 11.87	< 0.001
AST (u/L), median (Q1–Q3)	21.00 (18.00–25.00)	22.00 (19.00–28.00)	< 0.001
ALT(u/L), median (Q1–Q3)	18.00 (14.00–23.00)	23.00 (17.00–32.00)	< 0.001
Serum folate (nmol/L), median (Q1–Q3)	38.51 (26.27–55.72)	33.98 (24.01–50.06)	< 0.001

*NAFLD* Nonalcoholic Fatty Liver Disease, *PIR* Poverty Income Ratio, *IFG* Impaired Fasting Glycemia, *IGT* Impaired Glucose Tolerance, *BMI* Body Mass Index, *GGT* Gamma glutamyl transferase, *AST* Aspartate Transaminase, *ALT* Alanine Aminotransferase.

### Baseline characteristics of participants based on serum folate stratification

As shown in Table 2, all 5417 participants were divided into three groups according to serum folate tertile: low (1.8–12.6, n = 1806), middle (12.7–20.5, n = 1789) and high (20.6–49.9, n = 1822). Participants with middle or high serum folate had lower rates of NAFLD or AHF than participants with low serum folate. Participants with high serum folate were more likely to be older, female, non-Hispanic White, education level > high school, PIR > 3, never smokers, low work activity intensity, recreationally active, and lower waist circumference, or BMI, suggesting that this group of participants may have better economic status and lifestyle habits. Notably, we observed a higher percentage of participants with high serum folate who had hypertension or diabetes.

**Table 2:** Baseline characteristics of participants based on serum folate stratification.

Characteristic	Serum folate			p-value
	Low	Middle	High	
N	1806	1789	1822	
Demographics				
Age (year), mean ± SD	45.89 ± 16.20	47.74 ± 17.20	54.04 ± 17.38	< 0.001
Sex				< 0.001
Male	876 (48.50%)	851 (47.57%)	724 (39.74%)	
Female	930 (51.50%)	938 (52.43%)	1098 (60.26%)	
Race				< 0.001
Mexican American	245 (13.57%)	266 (14.87%)	219 (12.02%)	
Other Hispanic	170 (9.41%)	219 (12.24%)	176 (9.66%)	
Non-Hispanic White	628 (34.77%)	674 (37.67%)	910 (49.95%)	
Non-Hispanic Black	535 (29.62%)	339 (18.95%)	223 (12.24%)	
Other race—including multi-racial	228 (12.62%)	291 (16.27%)	294 (16.14%)	
Education level				< 0.001
< High school	398 (22.04%)	343 (19.17%)	327 (17.95%)	
High school	457 (25.30%)	379 (21.19%)	352 (19.32%)	
> High school	951 (52.66%)	1067 (59.64%)	1143 (62.73%)	
PIR				< 0.001
≤ 1	466 (25.80%)	368 (20.57%)	343 (18.83%)	
1–3	799 (44.24%)	778 (43.49%)	695 (38.14%)	
> 3	541 (29.96%)	643 (35.94%)	784 (43.03%)	
Behavioral characteristics				
Smoking status				< 0.001
Never	977 (54.10%)	1089 (60.87%)	1148 (63.01%)	
Now	449 (24.86%)	272 (15.20%)	188 (10.32%)	
Former	380 (21.04%)	428 (23.92%)	486 (26.67%)	
Work activities status				0.007
No	1015 (56.20%)	1047 (58.52%)	1063 (58.34%)	
Moderate	391 (21.65%)	388 (21.69%)	442 (24.26%)	
Vigorous	88 (4.87%)	61 (3.41%)	63 (3.46%)	
Both	312 (17.28%)	293 (16.38%)	254 (13.94%)	
Recreational activities status				< 0.001
No	991 (54.87%)	892 (49.86%)	825 (45.28%)	
Moderate	438 (24.25%)	460 (25.71%)	584 (32.05%)	
Vigorous	131 (7.25%)	136 (7.60%)	134 (7.35%)	
Both	246 (13.62%)	301 (16.83%)	279 (15.31%)	
Dietary characteristics				
Dietary energy intake (kcal), mean ± SD	3947.76 ± 1566.43	4024.92 ± 1575.39	3903.65 ± 1482.64	0.058
Dietary protein intake (mg), mean ± SD	155.46 ± 68.45	161.88 ± 67.71	157.49 ± 68.61	0.016
Dietary folate intake (mcg), median (Q1–Q3)	593.50 (433.00–837.00)	712.00 (505.00–975.00)	776.00 (543.00–1053.75)	< 0.001
Disease conditions				
NAFLD				< 0.001
No	930 (51.50%)	1020 (57.02%)	1106 (60.70%)	
Yes	876 (48.50%)	769 (42.98%)	716 (39.30%)	
AHF				0.937
No	1631 (90.31%)	1614 (90.22%)	1650 (90.56%)	
Yes	175 (9.69%)	175 (9.78%)	172 (9.44%)	
Hypertension status				< 0.001
No	1096 (60.69%)	1052 (58.80%)	989 (54.28%)	
Yes	710 (39.31%)	737 (41.20%)	833 (45.72%)	
Diabetes status				< 0.001
No	1166 (64.56%)	1107 (61.88%)	1078 (59.17%)	
Yes	325 (18.00%)	396 (22.14%)	419 (23.00%)	
IGT	127 (7.03%)	112 (6.26%)	165 (9.06%)	
IFG	188 (10.41%)	174 (9.73%)	160 (8.78%)	
Anthropometric measurements				
Waist circumference (cm), mean ± SD	102.31 ± 18.12	100.04 ± 17.10	97.96 ± 15.39	< 0.001
BMI (kg/m^2^), mean ± SD	30.66 ± 7.94	29.55 ± 7.06	28.39 ± 6.25	< 0.001
Biochemical indicators				
GGT (IU/L), median (Q1–Q3)	19.00 (14.00–29.00)	19.00 (14.00–28.00)	18.00 (13.00–26.00)	0.019
Triglycerides (mg/dL), median (Q1–Q3)	96.00 (65.00–146.00)	94.00 (64.00–138.00)	98.50 (67.00–141.00)	0.355
Total cholesterol (mg/dL), mean ± SD	189.26 ± 40.77	186.88 ± 40.62	190.97 ± 40.19	< 0.001
HDL cholesterol (mg/dL), mean ± SD	50.88 ± 14.10	53.05 ± 15.09	55.65 ± 15.75	< 0.001
AST (u/L), median (Q1–Q3)	21.00 (17.00–25.00)	22.00 (18.00–26.00)	23.00 (19.00–27.00)	0.002
ALT(u/L), median (Q1–Q3)	19.00 (14.00–27.00)	20.00 (15.00–28.00)	20.00 (16.00–26.00)	0.195

*NAFLD* Nonalcoholic fatty liver disease, *AHF* advanced hepatic fibrosis, *PIR* poverty income ratio, *IFG* impaired fasting glycemia, *IGT* impaired glucose tolerance, *BMI* body mass index, *GGT* gamma glutamyl transferase, *AST* aspartate transaminase, *ALT* alanine aminotransferase.

### Association of serum folate with NAFLD or AHF

Table 3 demonstrates the crude and adjusted odds ratios of serum folate with NAFLD and AHF. A negative association of serum folate with NAFLD was observed in all models. In the completely adjusted model (model 3), participants in the high serum folate group exhibited 27% lower odds of NAFLD in comparison to the low serum folate group (OR 0.73, 95% CI 0.62, 0.87, p = 0.0003), and a similar odds reduction was observed in the medium serum folate group. In addition, for each standard deviation increase in serum folate, the odds of NAFLD decreased by 15% in participants (OR 0.85, 95% CI 0.79, 0.91, p < 0.0001).

**Table 3:** Association of serum folate with NAFLD and AHF.

Models	NAFLD		AHF	
	OR (95% CI)	p-value	OR (95% CI)	p-value
Crude model				
Tertiles of serum folate				
Low	Ref		Ref	
Middle	0.80 (0.70, 0.91)	0.0009	1.01 (0.81, 1.26)	0.9258
High	0.69 (0.60, 0.78)	< 0.0001	0.97 (0.78, 1.21)	0.7982
Serum folate, per SD increase	0.85 (0.81, 0.90)	< 0.0001	1.06 (0.97, 1.15)	0.2294
Adjusted model 1				
Tertiles of serum folate				
Low	Ref		Ref	
Middle	0.80 (0.70, 0.91)	0.0011	0.86 (0.67, 1.10)	0.2283
High	0.65 (0.56, 0.75)	< 0.0001	0.50 (0.39, 0.64)	< 0.0001
Serum folate, per SD increase	0.82 (0.77, 0.87)	< 0.0001	0.76 (0.69, 0.84)	< 0.0001
Adjusted model 2				
Tertiles of serum folate				
Low	Ref		Ref	
Middle	0.84 (0.72, 0.98)	0.0314	0.67 (0.51, 0.88)	0.0039
High	0.73 (0.62, 0.86)	0.0002	0.47 (0.35, 0.62)	< 0.0001
Serum folate, per SD increase	0.85 (0.79, 0.91)	< 0.0001	0.77 (0.69, 0.85)	< 0.0001
Adjusted model 3				
Tertiles of serum folate				
Low	Ref		Ref	
Middle	0.83 (0.71, 0.98)	0.0254	0.66 (0.50, 0.87)	0.0037
High	0.73 (0.62, 0.87)	0.0003	0.47 (0.35, 0.63)	< 0.0001
Serum folate, per SD increase	0.85 (0.79, 0.91)	< 0.0001	0.77 (0.69, 0.86)	< 0.0001

*NAFLD* Nonalcoholic fatty liver disease, *AHF* advanced hepatic fibrosis, *PIR* poverty income ratio.

Crude model was not adjusted.

Adjusted model 1 adjusted for age, sex, race, education level, PIR.

Adjusted model 2 adjusted for model 1 + total cholesterol, HDL cholesterol, Hypertension status, Diabetes status.

Adjusted model 3 adjusted for model 2 + smoking status, work activities status, recreational activities status, dietary energy intake, dietary protein intake, dietary alcohol intake, dietary folate intake.

For AHF, no association was observed between serum folate and AHF in the crude model. In adjusted model 3, participants in the high serum folate group exhibited 53% lower odds of AHF than those in the low serum folate group (OR 0.47, 95% CI 0.35, 0.63, p < 0.0001). For each standard deviation increase in serum folate, the odds of AHF decreased by 23% in participants (OR 0.77, 95% CI 0.69, 0.86, p < 0.0001).

In addition, age, sex, race, education, PIR, smoking status, physical activity status, hypertension status, diabetes status, total cholesterol, HDL cholesterol, dietary protein intake, and dietary folate intake were significantly associated with NAFLD status in adjusted model 3. Age, sex, race, education, PIR, smoking status, physical activity status, hypertension status, diabetes status, total cholesterol, HDL cholesterol, and dietary folate intake were significantly associated with AHF status (Appendix Table 1).

**Figure 2:**
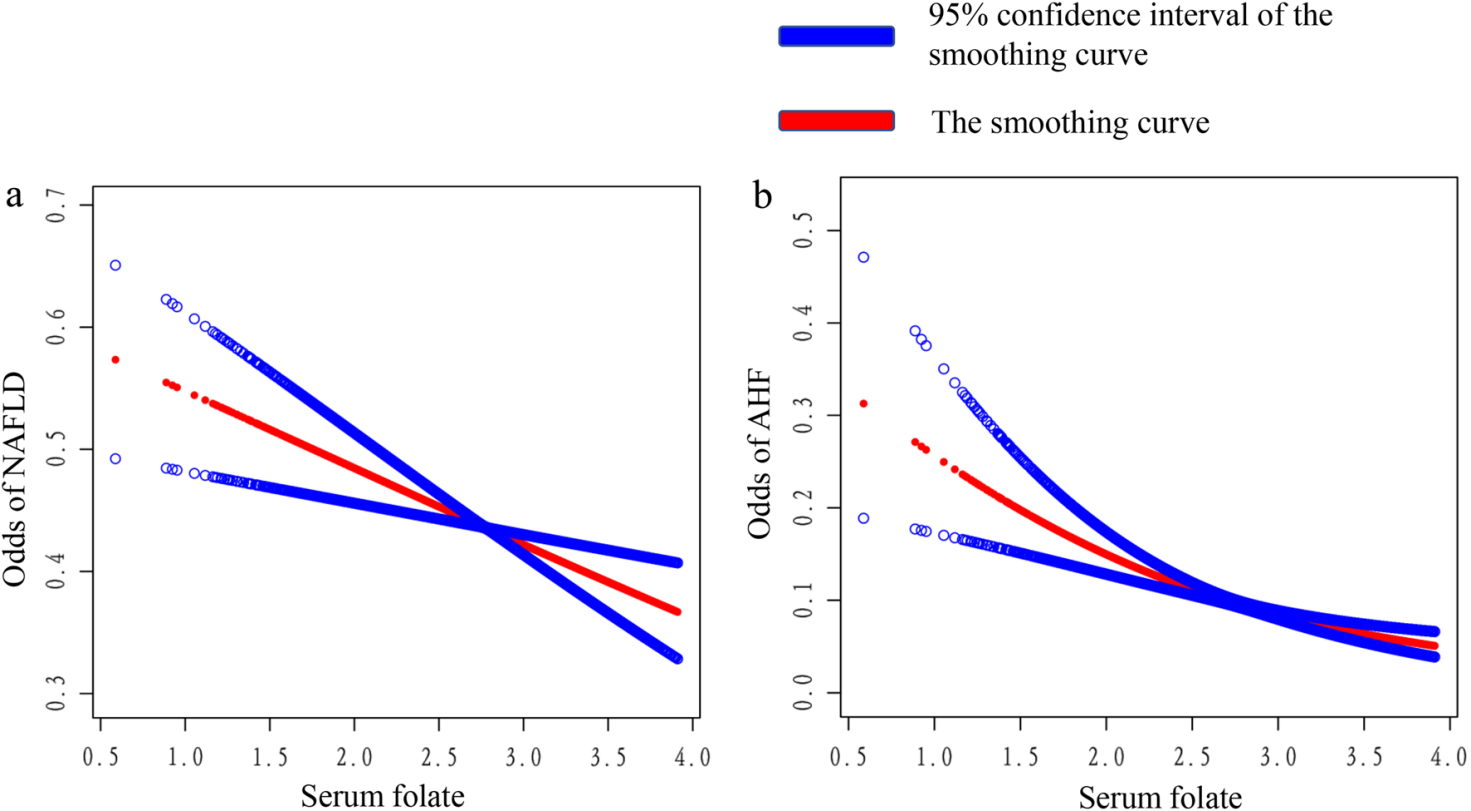
Smoothing curve fitting plot.

Figure 2 demonstrates a smoothed curve fit plot of the association, with serum folate showing a linear negative trend with both NAFLD and AHF.

### Propensity score matching

**Figure 3:**
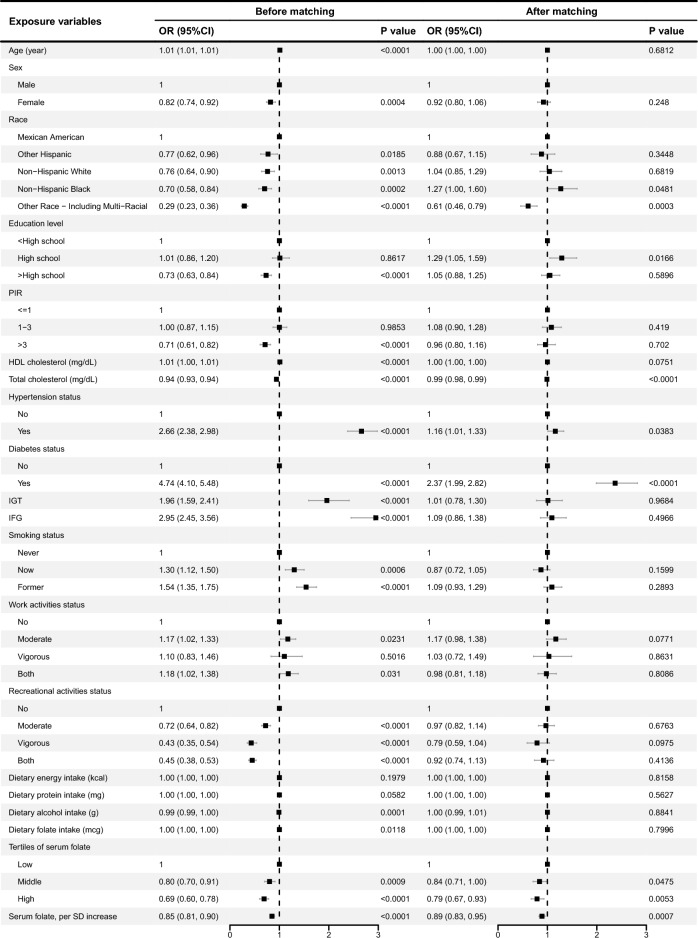
Multivariate analysis before and after matching for NAFLD.

A comparable control group constructed based on nearest neighbor propensity score matching (1:1) was used to further explore the association of serum folate with NAFLD and AHF. For NAFLD, 1640 participants were included in both the NAFLD and control groups after propensity score matching. Figure 3 shows the results of the multivariate analysis before and after matching. After matching, participants in middle and high serum folate group exhibited 16% (p = 0.0475) and 21% (p = 0.0053) lower odds of NAFLD in comparison to the low serum folate group, respectively. For each standard deviation increase in serum folate, the odds of NAFLD decreased by 11% in participants (OR 0.89, 95% CI 0.83, 0.95, p = 0.0007).

**Figure 4:**
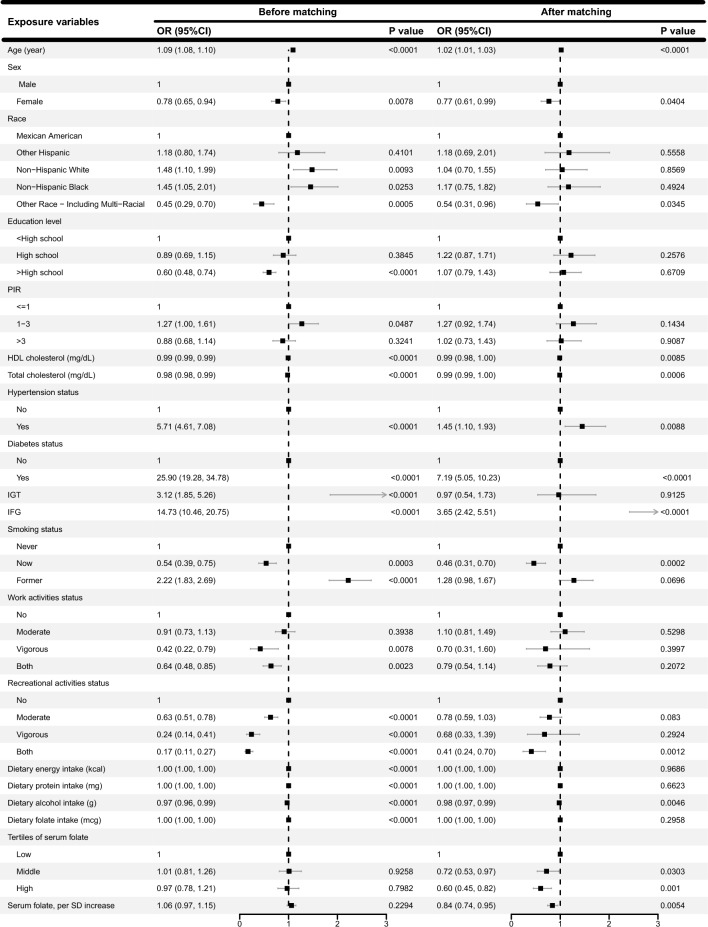
Multivariate analysis before and after matching for AHF.

For AHF, 519 participants were included in both the AHF and control groups after propensity score matching. Figure 4 shows the results of the multivariate analysis before and after matching. After matching, participants in middle and high serum folate group exhibited 28% (p = 0.0303) and 40% (p = 0.001) lower odds of AHF in comparison to the low serum folate group, respectively. For each standard deviation increase in serum folate, the odds of AHF decreased by 16% in participants (OR 0.84, 95% CI 0.74, 0.95, p = 0.0054).

### Stratified analysis

**Figure 5:**
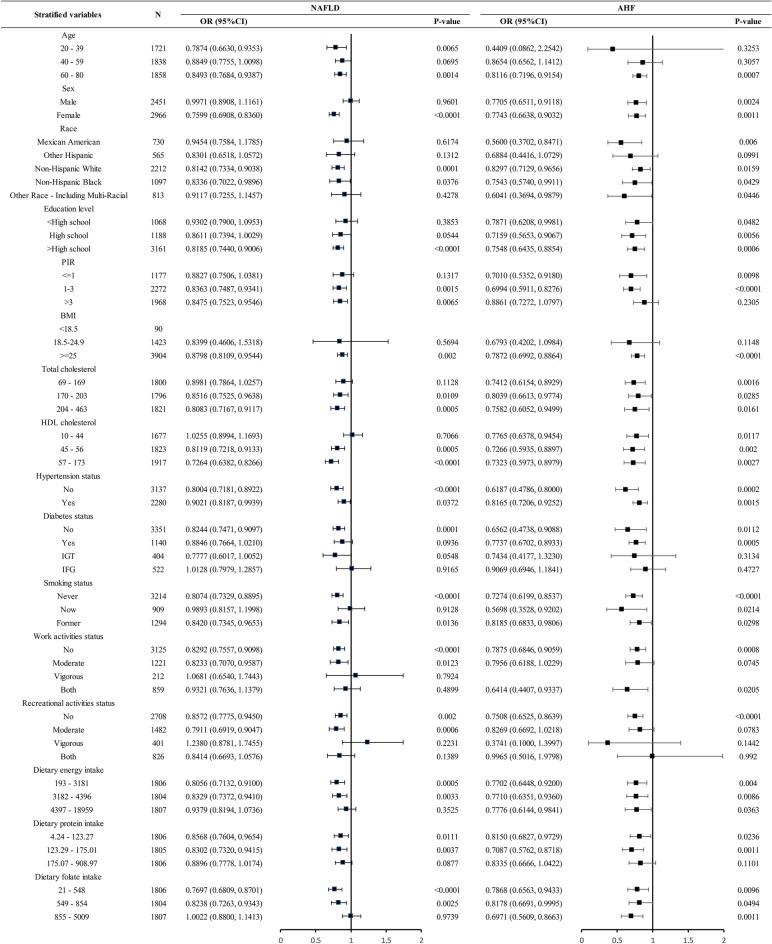
Stratified analysis. *NAFLD* Nonalcoholic fatty liver disease, *AHF* advanced liver fibrosis. The adjusted model in the stratification analysis was constructed based on model 3, adjusted for age, sex, race, education level, PIR, total cholesterol, HDL cholesterol, Hypertension status, Diabetes status, smoking status, work activities status, recreational activities status, dietary energy intake, dietary protein intake, dietary folate intake and dietary alcohol intake. Stratification variables were excluded from the adjusted model.

We constructed stratified analyses based on age, sex, race, education level, PIR, BMI, smoking status, work activities status, recreational activities status, dietary energy intake, dietary protein intake, dietary folate intake, hypertension status, diabetes status, total cholesterol, and HDL cholesterol. The results of the stratified analysis are shown in Fig. 5, and the negative correlation of serum folate with NAFLD and AHF exhibited a broad consistency across populations. No significant interaction was found in this study (p-interaction < 0.05).

## Discussion

This study analyzed NHANES data from 2011 to 2018 and elucidated the association between serum folate and NAFLD and AHF in US adults based on epidemiological studies for the first time. We found that higher serum folate level was associated with lower odds of NAFLD and AHF after controlling for confounding factors. Subsequently, a stratified analysis was conducted to explore the stability of the association across populations. The results of stratified analysis indicated that the association between serum folate and NAFLD and AHF exhibited excellent stability, with similar associations observed in almost all subgroups. Although results contradicting the findings were observed in a very small number of subgroups, none were statistically significant. There were no interactions for any of the covariates included in this study.

The association between folate and NAFLD is not the first time that attention has been drawn to it, so some of the previous relevant studies should not be overlooked. A randomized controlled trial of dietary intervention in Israel observed greater reductions in intrahepatic fat (IHF) in subjects with the most significant elevations in serum folate, suggesting that serum folate is effective in reducing the risk of developing NAFLD^20^. Mahamid et al. found that low folate levels were significantly associated with the severity of fibrosis^21^. The risk of NAFLD was negatively associated with serum folate in a recent meta-analysis^12^. The above findings were consistent with our study's conclusions. Nevertheless, two past studies based on US populations reached conclusions that contradict this study. Li Li et al. studied the association of vitamin B12 markers with NAFLD with data from NHANES 1999–2004 and claimed that serum folate was not associated with NAFLD^14^. Sources of inconsistency in the conclusions are the differences in year and adjustment models, and we added variables adjusting for physical activity, smoking, and dietary intake of the participants. Xiaohui Liu et al. researched the association between vitamins and NAFLD, but no association was found between dietary intake of folic acid and NAFLD^13^. We considered that differences in dietary intake of folic acid and serum folate level were the main reason for the different conclusions. Overall, we have strong confidence in the findings of this study due to the well-adjusted model, detailed stratification study and large sample size.

The current research on the possible mechanisms by which folic acid reduced the risk of NAFLD and AHF focused on improving abnormalities in lipid metabolism. Cellular AdoMet-dependent methylation reactions are required for the synthesis of phosphatidylcholine (PC), which is normally converted to triglycerides (TG)^22^. High levels of serum folate help to control or reduce AdoMet concentrations so that PC synthesis is inhibited to reduce the accumulation of triglycerides in the liver. Moreover, it has been shown that phosphatidylethanolamine (PE) is mediated by AdoMet via *N*-methyltransferase (PEMT) to accelerate PC synthesis, followed by the induction of hepatic steatosis^22^. In contrast, high serum folate levels can reduce AdoMet concentrations and in turn inhibit the above PC synthesis pathway, ultimately improving abnormalities in hepatic lipid metabolism^23^. The protective effect of folic acid against oxidative stress in hepatocytes may also be a potential mechanism^24^. Serum folate promoted mitochondrial beta oxidation, reduced oxidative stress in vivo and inhibited peroxisome proliferator-activated receptor γ (PPARγ). PPARγ is the key factor in regulating adipogenesis and decreasing TG accumulation in the liver, thus reducing hepatic steatosis^25^. In addition, the severity of NAFLD and the progression of AHF were associated with higher systemic levels of some cytokines, such as IL-6 and TNF-α^26^. High serum folate levels can help to reduce the expression of proinflammatory cytokines and inhibit the recruitment and activation of Kupffer cells, thereby lowering the risk of NAFLD and AHF^27,28^.

In this study, age, sex, race, education, PIR, smoking status, physical activity status, hypertension status, diabetes status, total cholesterol, HDL cholesterol, dietary protein intake and dietary folate intake were significantly associated with NAFLD status. Previous studies have shown that hypertension and diabetes are important risk factors for NAFLD^29^. Smoking is positively associated with NAFLD and the underlying mechanisms have been initially elucidated^30,31^. The association between physical activity status and NAFLD has also been previously reported, with exercise helping to reduce functional adaptations in patients with NAFLD^32,33^. In addition, race, education, and PIR are important social determinants of NAFLD^34–36^. Of note, dietary folate intake was significantly associated with NAFLD and AHF. Participants with high dietary folate intake had 20% lower odds of suffering from NAFLD (OR 0.80, 95% CI 0.66, 0.98, p = 0.031) and 36% lower odds of suffering from AHF (OR 0.64, 95% CI 0.46, 0.90, p = 0.0109) compared to participants with low dietary folate intake. The intake of dietary folate is a significant source of serum folate supplementation. The association between dietary folate and both NAFLD and AHF provides partial support for the findings of this study.

Notably, this study observed significant sex differences in the correlation between serum folate and NAFLD, which was lacking in previous studies. One possible explanation may be that higher levels of estrogen in women exerted a protective effect. A study by Nemoto et al. found that estrogen supplementation prevented the progression of hepatic steatosis adenopathy in estrogen-deficient mice, suggesting that estrogen receptor-mediated signaling pathways may play a key role in lipid metabolism in the liver^37,38^. Additionally, Kupffer cells in men expressed higher levels of TLR4 than those in women to the extent that they produced more proinflammatory cytokines, further activating liver inflammation and fibrosis^39^. Unlike men, Kupffer cells in women exhibited more anti-inflammatory and anti-fibrotic properties.

A highlight of this study is the larger and scientifically designed sample source, which enhanced the credibility and universality of the findings. In addition, the well-established adjustment model and stratified analysis make the conclusions more reliable. However, there are still some limitations of our study that cannot be ignored. First, due to the nature of cross-sectional studies, we cannot establish a causal relationship between serum folate and NAFLD and AHF, and further prospective cohort studies are necessary. Second, although we included as many covariates as possible to exclude bias from confounding factors, there may still be potential confounders that were not included in the analysis. Third, all participants in this study were from the United States, and the applicability of the results to populations in other countries needs to be carefully considered, given the differences in physical condition, dietary habits and environmental factors that exist between populations. In addition, although FLI showed a high diagnostic value, it is not a substitute for biopsy. The diagnosis of NAFLD in this study is not a clinical diagnosis, and further studies are still needed in the future. Overall, despite the strong statistical efficacy of this study, there is a requirement for greater modesty and caution in interpreting the results due to the limitations of cross-sectional studies as well as the diagnosis of NAFLD.

## Conclusions

The results of this study indicate that higher serum folate level was associated with lower odds of NAFLD and AHF among US adults. Future prospective cohort studies are still necessary to validate our conclusions.

### Supplementary Information

Supplementary Information.

## Data Availability

Original data generated and analyzed during this study are included in this published article or in the data repositories listed in References. The dataset supporting the conclusions of this article is available in the NHANES repository, https://www.cdc.gov/nchs/nhanes/index.htm.
